# Curcumin Inhibits HGF-Induced EMT by Regulating c-MET-Dependent PI3K/Akt/mTOR Signaling Pathways in Meningioma

**DOI:** 10.1155/2021/5574555

**Published:** 2021-08-09

**Authors:** Xiaodong Chen, Fen Tian, Peng Lun, Yugong Feng

**Affiliations:** ^1^Department of Neurosurgery, The Affilatied Hospital of Qingdao University, Qingdao, Shandong 266003, China; ^2^Department of Nephrology, The Affilatied Hospital of Qingdao University, Qingdao, Shandong 266003, China

## Abstract

Meningiomas, which are the most common primary intracranial tumors, have highly aggressive cells in malignant cases. Due to its extensive antitumor effects, curcumin is widely used in experimental and clinical studies. However, the role of curcumin during the epithelial-mesenchymal transition (EMT) in meningioma has not been established. We found that curcumin blocks hepatocyte growth factor- (HGF-) induced proliferation, migration, invasion, and EMT of human malignant meningioma cells by regulating the PI3K/Akt/mTOR signaling pathway. In addition, treatment of human malignant meningioma cells with the tyrosine protein kinase (c-MET) inhibitor (SU11274) or the phosphoinositide 3-kinase (PI3K) inhibitor (LY294002) suppressed HGF-induced migration and EMT. Furthermore, we found that curcumin inhibited tumor growth and HGF-induced EMT in mice subjected to subcutaneous xenotransplantation. These findings indicate that HGF regulates EMT in human malignant meningioma cells through c-MET/PI3K/Akt/mTOR modulation. In conclusion, curcumin inhibits HGF-induced EMT by targeting c-MET and subsequently blocking the PI3K/Akt/mTOR pathway.

## 1. Introduction

Meningiomas, which are the most common extracranial tumors of the brain, originate from arachnoidal cap cells of the leptomeninges. In 2016, the World Health Organization (WHO) classified meningiomas into WHO grade I (benign), WHO grade II (atypical), and WHO grade III (malignant) [1]. WHO grade I refers to a benign meningioma that usually recovers after surgical treatment and has a good prognosis. WHO grade II and WHO grade III meningiomas are rare, more invasive, and metastatic, and they also have a higher recurrence rate [1]. Traditional chemotherapy, radiotherapy, and biological immunotherapy are ineffective in the treatment of meningioma and are associated with significant sequelae [2]. Therefore, there is an urgent need to develop new therapeutic drugs and explore new intervention targets.

Epithelial-mesenchymal transition (EMT), whose activation is related with tumor development and metastasis, is associated with high-grade and recurrent meningiomas [3]. For tumor cells undergoing the EMT process, epithelial cells lose polarity and intercellular as well as adhesion junctions and exhibit mesenchymal cell morphologies and characteristics, thereby gaining infiltration, migration, and invasion abilities [3]. When EMT occurs, expression levels of epithelial markers, including E-cadherin and keratin, are downregulated, while the expression levels of mesenchymal markers such as N-cadherin, vimentin, and fibronectin are upregulated. Growth factors in the extracellular matrix bind specific receptors on the cell membrane, upregulate, and activate EMT transcription factors, thereby activating the EMT process [4]. Hepatocyte growth factor (HGF) is a growth factor that binds to its specific receptor, tyrosine protein kinase (c-MET), and facilitates EMT progression by activating c-MET, which in turn activates multiple downstream signaling pathways. In addition, HGF and c-MET are overexpressed in meningioma and are predictive markers as well as targeted therapy molecules for meningioma [5]. The HGF/c-MET signaling pathway regulates downstream PI3K/Akt/mTOR, Ras/MAPK, and JAK/STAT signaling pathways, which are important in regulating tumor cell growth, migration, and invasion [6,7]. Moreover, PI3K/Akt/mTOR signaling pathway abnormalities have been reported in breast, liver, lung, and prostate cancers [8–11]. Therefore, the PI3K/Akt/mTOR signaling pathway has become a focal point for cancer therapy and targeted drug development [12]. The PI3K/Akt/mTOR signaling pathway has been shown to be activated in aggressive meningioma, while mTOR inhibitors were found to suppress meningioma tumor cell proliferation [13, 14]. The PI3K/Akt/mTOR signaling pathway is, thus, regarded as a potential therapeutic target.

Recently, traditional Chinese medicine has been applied as an adjuvant therapy after tumor radiotherapy and chemotherapy. Curcumin, a natural fat-soluble phenolic compound that is extracted from rhizomes of turmeric (*Curcuma longa* L.), has anti-inflammatory, antioxidant, and anticancer properties. Moreover, it is safe and effective [15]. As an anti-inflammatory herbal agent, curcumin was found to alleviate inflammation after radiotherapy and chemotherapy [16]. Anticancer effects of curcumin, including chemopreventive, chemotherapeutic, and tumor preventive effects, have also been evaluated [17]. Curcumin has definite anticancer properties with low cytotoxic effects [17]. A previous study reported that curcumin exerts antitumorigenic effect on meningioma cells. Furthermore, curcumin is indirectly involved in EMT by regulating the Wnt, PI3K-Akt, NF-*κ*B, and MAPK/ERK signaling pathways, thereby regulating tumor cell proliferation, migration, and invasion of cancer cells [18, 19]. These findings imply that antitumorigenic effects of curcumin may be attributed to the inhibition of tumor cell proliferation and EMT.

This study aimed at determining whether curcumin inhibits HGF-induced EMT of meningioma cells. We found that curcumin inhibited HGF-induced EMT through the PI3K/Akt/mTOR signaling pathway.

## 2. Materials and Methods

### 2.1. Sample Collection

Between March 2016 and March 2019, tumor tissues from 15 benign (WHO grade I) and 10 malignant (WHO grade III) patients with pathologically confirmed meningiomas were collected. Ethical approval for this study was obtained from the Ethical Committee of The Affiliated Hospital of Qingdao University (no. QYFYWZLL26075). Written informed consent was obtained from all patients included in the study.

### 2.2. Cell Culture

The human malignant meningioma cell line (IOMM-Lee) was purchased from the Chinese Academy of Sciences (Beijing, China). IOMM-Lee cells were plated at a density of 1 × 10^5^ cells per well in six plates and maintained in Dulbecco's Modified Eagle Medium (DMEM) (Sigma, China) supplemented with 10% fetal bovine serum (FBS) and incubated in a 5% CO_2_ humidified atmosphere at 37°C for 24 h.

Optimal inhibitory concentrations of curcumin on IOMM-Lee cells were determined by seeding 2 × 10^3^ cells in 96-well plates with FBS-free DMEM and incubated for 24 h. Then, cells were divided into 10 groups and separately incubated with a series of concentrations (5, 10, 20, 40, 60, 80, and 100 *μ*mol/l) of curcumin (Sigma, China) or DMSO (Sigma, China) in a 5% CO_2_ humidified atmosphere at 37°C. After 24, 48, and 72 h, the CCK8 assay was performed to detect the inhibitory effects of curcumin. In brief, the CCK8 solution was incubated with cells at 37°C after which absorbance at 450 nm was determined after 2 h. Then, cells were divided into the control and curcumin groups (IOMM-Lee cells incubated with DMSO or 10, 20, and 30 *μ*mol/l curcumin for 24 h, respectively, and stimulated by 50 ng/m HGF for 24 h) as well as the signaling pathway inhibitor group (IOMM-Lee cells stimulated with 50 ng/mL HGF for 24 h and then treatment with 5 *μ*mol/l c-MET inhibitor SU11274 or 25 *μ*mol/l PI3K inhibitor LY294002 for 24 h).

### 2.3. Immunohistochemistry

Expression levels and locations of HGF, c-MET, E-cadherin, N-cadherin, and vimentin were analyzed by immunohistochemistry. In brief, meningioma tissues were fixed in 4% paraformaldehyde and later embedded in paraffin for immunohistochemistry. *μ*m tissue sections were deparaffinized and dehydrated after which antigen retrieval was performed by microwave treatment in citrate buffer for 15 minutes. To inhibit the potential occurrence of false positive results, blocking with 3% H_2_O_2_/methanol for 10 min was followed by blocking with goat serum for 20 min. Sections were incubated with primary antibodies (anti-HGF, 1 : 500, ab216623; anti-c-MET, 1 : 1000, ab137654; anti-E-cadherin, 1 : 1000, ab231303; anti-N-cadherin, 1 : 5000, ab76011; Abcam, China) for 1 h at 25°C, respectively. Then, they were incubated with biotinylated anti-rabbit/mouse IgG and peroxidase-labeled streptavidin for 10 min, respectively.

### 2.4. Flow Cytometry Analysis

Cell apoptosis was determined by flow cytometry analysis. The IOMM-Lee cells were seeded into 6-well plates and starved for 24 h after which they were treated with a binding buffer and washed twice using PBS. Then, they were stained with 5 *μ*l annexin V-FITC and 5 *μ*l propidium iodide (PI) for 15 min in the dark and analyzed by flow cytometry.

### 2.5. Western Blot

Western blot was performed to detect protein expression levels. Total proteins were extracted from cells and tissues using the RIPA buffer. Protein lysates were centrifuged at 4°C for 40 min after which protein concentrations in the supernatant were determined using the bicinchoninic acid solution (BCA) protein assay kit (Thermo Fisher Scientific, China). Proteins were separated by 12% sodium dodecyl sulfate-polyacrylamide gel electrophoresis (SDS-PAGE) and transferred to polyvinylidene fluoride (PVDF) membranes. Membranes were blocked with 5% nonfat milk powder at room temperature for 2 h. Then, they were incubated with primary antibodies (anti-c-MET, 1 : 1000, ab137654; anti-p-c-MET, 1 : 1000, ab68141; anti-Akt, 1 : 500, ab8805; anti-p-Akt, 1 : 1000, ab38449; anti-mTOR, 1 : 1000, ab32028; anti-p-mTOR, 1 : 10000, ab134903; anti-E-cadherin, 1 : 1000, ab231303; anti-N-cadherin, 1 : 5000, ab76011; anti-vimentin, 1 : 5000, ab137321; Abcam, China) at 4°C overnight. On the next day, membranes were washed using TBST and incubated with secondary antibodies at room temperature for 1 h. Immunoblots were detected using the enhanced chemiluminescence system (Millipore, Darmstadt, Germany).

### 2.6. Cell Counting Kit-8 (CCK8) Assay

Cell proliferation abilities were determined using the CCK8 assay. IOMM-Lee cells (2 × 10^4^ cells/well) were seeded in 96-well plates overnight and starved for 24 h. Then, the CCK8 solution (10 *μ*L) was added into each well and incubated for 2 h at room temperature. Finally, absorbance was measured at 450 nm.

### 2.7. Migration and Invasion Assays

Migration and invasion abilities of the IOMM-Lee cells were determined through wound healing and transwell assays, respectively. For the wound healing assay, 4 × 10^4^ IOMM-Lee cells were seeded in 6-well plates with Ibidi culture inserts (Ibidi^®^, Martinsried, Germany). Wound imaging was performed at 0 and 24 h. For the invasion assay, 4 × 10^5^ IOMM-Lee cells were seeded in the upper chamber of transwells, while lower chambers were filled with 600 *µ*l of DMEM containing 2% FBS as a chemotactic factor. Then, they were incubated in a 5% CO_2_ humidified atmosphere at 37°C for 24 h. Cells in the lower chambers were fixed with 4% paraformaldehyde, stained with crystal violet, imaged, and counted under a microscope.

### 2.8. Animal Studies

Animal experiments were approved by the Ethical Committee of The Affiliated Hospital of Qingdao University. C57BL/6 nude mice were purchased from Qingdao University (Shandong, China). Subcutaneous xenotransplantation models were established by subcutaneously injecting 100 *μ*l of IOMM-Lee cells (1 × 10^7^) into the right flanks of 4-5-week-old C57BL/6 nude mice. Fifteen days after injection, nude mice were allocated into 4 groups: the ctrl group (*n* = 4), HGF group (*n* = 4), HGF + 100 mg/kg curcumin group, and HGF + 300 mg/kg curcumin group (*n* = 4). In brief, 30 *µ*g/kg HGF or PBS was injected into the tumor every 3 days. Moreover, 100 or 300 mg/kg curcumin was intraperitoneally administered every day. After 30 days of treatment, mice were euthanized by dislocating the cervical vertebrae. The transplanted tumors were resected and weighed. To detect the expression levels of proteins in the tumors, immunohistochemistry was performed.

### 2.9. Statistical Analysis

Data were expressed as mean ± standard deviation (SD) and analyzed by GraphPad Prism 9.0. Student's *t*-test and one-way analysis of variance (ANOVA) followed by Tukey's post hoc tests were used to analyze statistical differences between groups and among groups, respectively. *P* *≤* 0.05 was set as the threshold for statistical significance.

## 3. Results

### 3.1. Expression Levels of HGF and c-MET in Human Benign Meningioma Tissues Were Suppressed

**Figure 1:**
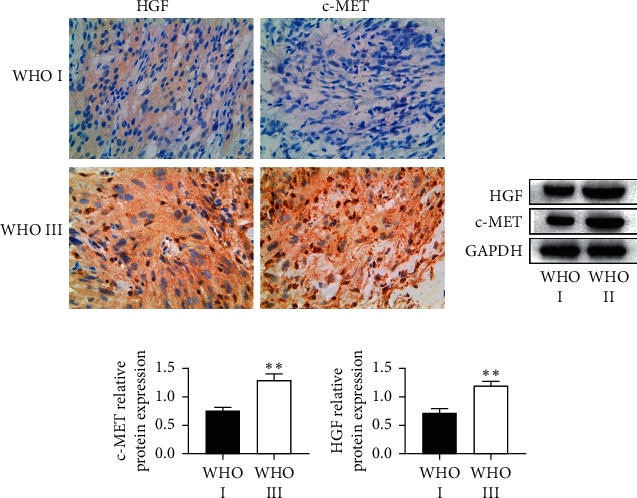
The expression of hepatocyte growth factor (HGF) and c-MET in the clinical tumor tissue of malignant (WHO grade III) and benign (WHO grade I) meningiomas. (a) The expression and location of HGF and c-MET were detected by immunohistochemistry staining (magnification: 400x). (b) The expression of HGF and c-MET was detected by western blot. (c) Statistical analysis result of c-MET expression. (d) Statistical analysis result of HGF expression. Data are expressed as mean ± standard error; the asterisks indicate significant differences (^*∗∗*^*P* < 0.01) between WHO I (*n* = 10) and WHO III (*n* = 10) meningiomas.

Expressions of HGF and c-MET have been associated with aggressive meningiomas [20]. To detect the expression of HGF and c-MET in meningioma, we performed immunohistochemistry and western blot assays. HGF and c-MET were found to be positively expressed in both benign and malignant meningiomas and were both localized in the cell membrane and cytoplasm. Compared to WHO grade I meningioma tissues, expression levels of HGF and c-MET were found to be elevated in WHO grade III (malignant) meningioma tissues (Figure 1(a)). Both HGF and c-MET were strongly positive in malignant meningioma tissues and weakly positive in benign meningioma tissues. To further investigate the expression of HGF and c-MET in benign and malignant meningiomas, western blot was performed in clinical tissues. It was found that HGF and c-MET were significantly upregulated in malignant meningioma compared with benign meningioma (Figures 1(b) and 1(c)). These results indicate that upregulations of HGF and c-MET are associated with the degree of malignancy of meningioma.

### 3.2. Curcumin Blocked the Proliferation of the Human Malignant Meningioma Cells

**Figure 2:**
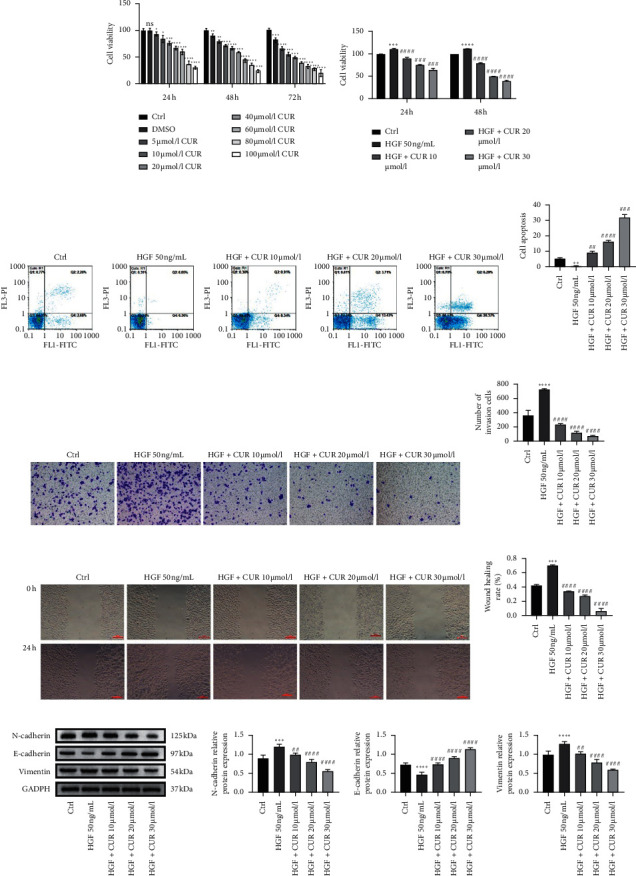
The effects of curcumin on HGF-induced cell proliferation, apoptosis, invasion, migration, and epithelial-mesenchymal transition of meningioma IOMM-Lee cells. (a) IOMM-Lee cells were starved for 12 hours and then stimulated by 50 ng/ml of HGF in the presence of 2% FBS for 24, 48, and 72 hours. (b) IOMM-Lee cells were starved for 12 hours and then stimulated by 50 ng/ml of HGF in the presence of 2% FBS, and curcumin was added 4 hours before stimulation. Cell proliferation was detected by the CCK-8 assay at 24 and 48 hours after HGF stimulation. (c) IOMM-Lee cells were starved for 12 hours and then stimulated by 50 ng/ml of HGF in the presence of 2% FBS, and curcumin was added 4 hours before stimulation. Cell apoptosis was evaluated by the flow cytometry assay. (d) Statistical analysis result of apoptosis. The asterisks indicate significant differences. (e) IOMM-Lee cells were seeded into the upper chamber without FBS and invaded toward the lower chamber containing 2% FBS and 50 ng/ml HGF in the media (magnification: 100x). (f) Statistical analysis result of invasion. The asterisks indicate significant differences. (g) IOMM-Lee cells were starved for 12 hours and then stimulated by 50 ng/ml of HGF (with 2% FBS). Cell migration capability was determined by the wound healing assay (magnification: 100x). (h) Statistical analysis result of migration; the asterisks indicate significant differences. (i) IOMM-Lee cells were starved for 12 hours and then stimulated by 50 ng/ml of HGF (with 2% FBS). The expression of E-cadherin, N-cadherin, and vimentin was detected by western blot. (j) Statistical analysis result of E-cadherin, N-cadherin, and vimentin expression. Data are expressed as mean ± standard error; *n* = 3; the asterisk (^*∗*^) indicates a significant difference compared to the ctrl group, ^*∗*^*P* < 0.05, ^*∗∗*^*P* < 0.01, ^*∗∗∗*^*P* < 0.001, and ^*∗∗∗∗*^*P* < 0.0001. The hash (#) indicates a significant difference compared to the HGF group, ^#^*P* < 0.05, ^##^*P* < 0.01, ^###^*P* < 0.001, and ^####^*P* < 0.0001.

Curcumin has been shown to induce apoptosis in meningioma [21]. To investigate the inhibitory effects of curcumin on the human malignant meningioma IOMM-Lee cell line, we first screened the optimal inhibitory concentrations of curcumin on IOMM-Lee cells. Specifically, IOMM-Lee cells were treated with different concentrations of curcumin after which cell viabilities were assessed through the CCK-8 assay at 24, 48, and 72 hours of incubation.  Figure 2(a) shows that curcumin inhibited the proliferation of IOMM-Lee cells. Cell proliferation was negatively correlated with curcumin in a concentration-dependent manner. In addition, curcumin at 10 to 30 *μ*mol/l significantly inhibited cell proliferation. Therefore, curcumin concentrations at 10, 20, and 30 *μ*mol/l for 24 h treatments were used in subsequent experiments.

### 3.3. Curcumin Inhibited HGF-Induced Human Malignant Meningioma Cell Proliferation

HGF induces the metastatic progression of different cells [22]. We have already shown that curcumin inhibits the proliferation of IOMM-Lee cells. To determine the effect of curcumin on HGF-induced IOMM-Lee cell proliferation, IOMM-Lee cells were treated with 50 ng/mL HGF for 24 h and different concentrations of curcumin (0, 10, 20, and 30 *μ*mol/l) for 24, 48, and 72 h. Then, the CCK-8 assay was performed.  Figure 2(b) shows that curcumin suppressed the proliferation of IOMM-Lee cells, and the maximum effect concentration was 30 *μ*mol/l. Moreover, flow cytometry revealed that curcumin enhanced the apoptosis of IOMM-Lee cells, and the maximum effect concentration was 30 *μ*mol/l (Figures 2(c) and 2(d)). These findings imply that curcumin inhibited the HGF-induced proliferation of IOMM-Lee cells.

### 3.4. Curcumin Inhibited HGF-Induced Human Malignant Meningioma Cell Migration and Invasion

Curcumin has been shown to inhibit HGF-induced tumor metastasis and invasion [17, 21]. To determine whether curcumin inhibited HGF-induced tumor metastasis and invasion of IOMM-Lee cells, cell wound healing and transwell invasion assays were performed. Compared to control cells, HGF stimulation enhanced the migration and invasion of IOMM-Lee cells. Curcumin treatment inhibited HGF-induced migration and invasion in a concentration-dependent manner (Figures 2(e) and 2(h)). These findings imply that curcumin inhibits the migration and invasion of meningioma cells.

### 3.5. Curcumin Inhibited HGF-Induced Human Malignant Meningioma Cell EMT

Migration and invasion have been associated with EMT, and HGF induces EMT [23]. To confirm the effect of curcumin on EMT in meningioma, we used western blot to determine the expression of EMT marker proteins (E-cadherin, N-cadherin, and vimentin). It was found that HGF suppressed protein expression levels of E-cadherin and elevated protein expression levels of N-cadherin and vimentin in IOMM-Lee cells (Figures 2(i) and 2(j)). However, curcumin reversed these effects in a concentration-dependent manner. Therefore, curcumin inhibits HGF-induced EMT of meningioma cells.

### 3.6. Curcumin Inhibited HGF-Induced EMT by Regulating c-MET-Dependent PI3K/Akt/mTOR Signaling Pathways

**Figure 3:**
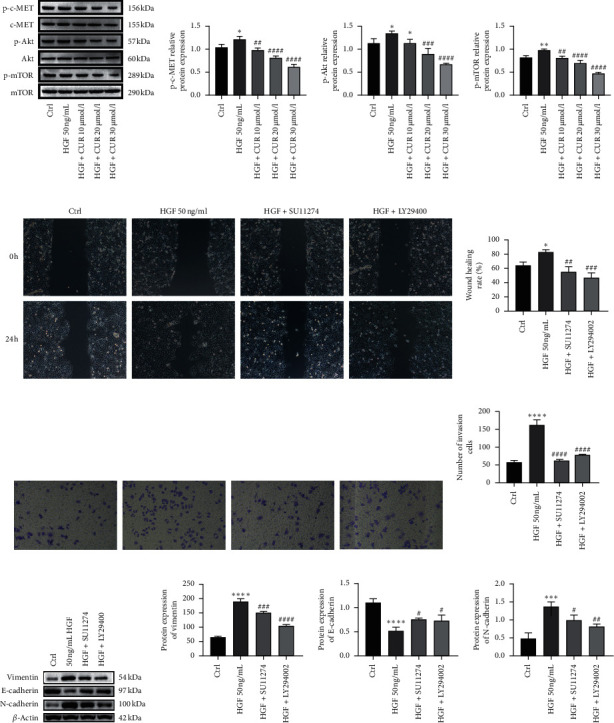
Curcumin inhibits HGF-induced EMT via modulation of c-MET/PI3K/Akt/mTOR signaling pathways. (a) IOMM-Lee cells were starved for 12 hours and then stimulated by 50 ng/ml of HGF in the presence of 2% FBS, and curcumin was added 4 hours before stimulation. The protein expression of the c-MET/PI3K/Akt/mTOR marker was detected by western blot. (b) Statistical analysis result of c-MET/PI3K/Akt/mTOR marker protein expression. (c) IOMM-Lee cells were starved for 12 hours and then stimulated by 50 ng/ml of HGF in the presence of 2% FBS, and MET inhibitor SU11274 (5 *μ*mol/l) or PI3K inhibitor LY294002 (25 *μ*mol/l) was used 4 hours before HGF stimulation (magnification: 100x). (d) Statistical analysis result of the wound healing assay. (e) IOMM-Lee cells were seeded into the upper chamber without FBS and invaded toward the lower chamber containing 2% FBS and 50 ng/ml HGF in the media. MET inhibitor SU11274 (5 *μ*mol/l) or PI3K inhibitor LY294002 (25 *μ*mol/l) was used 4 hours before HGF stimulation (magnification: 100x). (f) Statistical analysis result of invasion. (g) IOMM-Lee cells were starved for 12 hours and then stimulated by 50 ng/ml of HGF in the presence of 2% FBS, and MET inhibitor SU11274 (5 *μ*mol/l) or PI3K inhibitor LY294002 (25 *μ*mol/l) was used 4 hours before HGF stimulation. (h) Statistical analysis result of invasion. The protein expression of EMT markers, i.e., vimentin, E-cadherin, and N-cadherin, was detected by western blot. Data are expressed as mean ± standard error; *n* = 3; the asterisk (^*∗*^) indicates a significant difference compared to the ctrl group, ^*∗*^*P* < 0.05, ^*∗∗*^*P* < 0.01, ^*∗∗∗*^*P* < 0.001, and ^*∗∗∗∗*^*P* < 0.0001. The hash (#) indicates a significant difference compared to the HGF group, ^#^*P* < 0.05, ^##^*P* < 0.01, ^###^*P* < 0.001, and ^####^*P* < 0.0001.

c-MET is the receptor of HGF. Binding of HGF to c-MET activates the tyrosine kinase activity of c-MET, which promotes the proliferation, metastasis, and invasion of various tumor cells [23]. The PI3K/Akt/mTOR pathway is one of the major signaling pathways downstream of c-MET activation and is associated with cell metastasis, invasion, and EMT of cancer cells, including meningioma cells [13]. To determine whether HGF activated the c-MET/PI3K/Akt/mTOR signaling pathways in IOMM-Lee cells, western blot was performed to detect the expression of c-MET, Akt, and mTOR and their phosphorylation levels. Phosphorylation levels of c-MET, Akt, and mTOR were significantly increased in the HGF group compared with the control group (Figures 3(a) and 3(b)). Treatment of HGF-induced IOMM-Lee cells with curcumin and the phosphorylation levels of c-MET, Akt, and mTOR were decreased in a concentration-dependent manner (Figures 3(a) and 3(b)). These results indicated that HGF activated c-MET/PI3K/Akt/mTOR signaling pathways in IOMM-Lee cells, while curcumin reversed the activation in a concentration-dependent manner.

To further investigate the effects of the PI3K/Akt/mTOR pathway on HGF-induced meningioma cells, we treated IOMM-Lee cells with a c-MET inhibitor (SU11274, 5 *μ*mol/l) and a PI3K inhibitor (LY29400225, *μ*mol/l), respectively, and then the proliferation, migration, invasion, and EMT were detected. Figures 3(c) and 3(d) show that compared with the control group, HGF induced cell migration, whereas the two inhibitors reversed the HGF-induced cell migration. Similarly (Figures 3(e) and 3(f)), HGF induced the cell invasion of IOMM-Lee cells, while the two inhibitors reversed the HGF-induced invasion. In addition, we determined the expression levels of EMT marker proteins (E-cadherin, N-cadherin, and vimentin). Compared to the control group, the expression levels of E-cadherin were significantly downregulated in the HGF group, whereas the expression of N-cadherin and vimentin was significantly upregulated (Figures 3(g) and 3(h)). The two inhibitors elevated, the expression of E-cadherin was increased, and also the expression of N-cadherin and vimentin was decreased. Therefore, the results indicated that two inhibitors reversed HGF-induced EMT of meningioma cells. Taken together, curcumin inhibits HGF-induced EMT through the c-MET/PI3K/Akt/mTOR signaling pathway.

### 3.7. Curcumin Inhibited Tumor Growth and Metastasis in Tumor Xenografts

**Figure 4:**
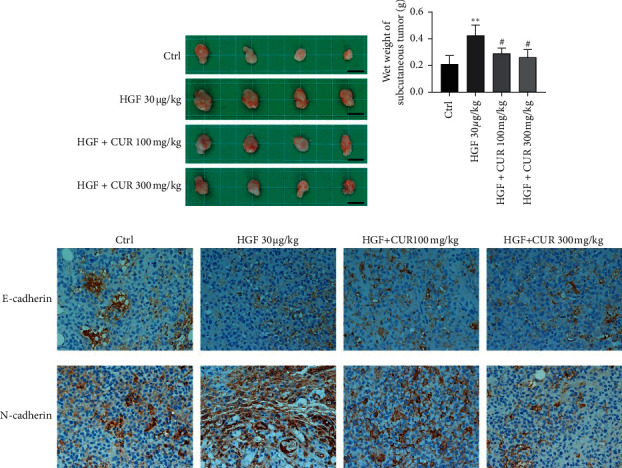
Curcumin effectively inhibited hepatocyte growth factor-induced tumor growth and epithelial-mesenchymal transition. (a) Image of tumor size (scale bar: 50 *µ*m). (b) Wet tumor weight at the time of dissection. (c) Immunohistochemistry (IHC) of the expression pattern of E-cadherin and N-cadherin of tumor tissues from the xenograft tumor model (magnification: 400x). Data are expressed as mean ± standard error; *n* = 4; the asterisk (^*∗*^) indicates a significant difference compared to the ctrl group, ^*∗∗*^*P* < 0.01. The hash (#) indicates a significant difference compared to the HGF group, ^#^*P* < 0.05.

To determine whether curcumin exerts inhibitory effects on tumorigenesis and EMT *in vivo*, we used a nude mouse xenograft model. We found that HGF induced an increase in tumor size and weight, while curcumin inhibited tumor growth in a concentration-dependent manner (Figures 4(a) and 4(b)). At the same time, EMT marker proteins were detected by immunohistochemistry. Compared to the control group, expression levels of E-cadherin were downregulated, while expression levels of N-cadherin were upregulated in the HGF group, implying that HGF induced EMT *in vivo* (Figure 4(c)). Curcumin suppressed the HGF-induced EMT in a concentration-dependent manner (Figure 4(c)). These results indicate that curcumin inhibits HGF-induced meningioma growth by suppressing EMT.

## 4. Discussion

Studies have reported that HGF is linearly correlated with the EMT process, and it accelerates tumorigenesis [24], including in meningioma [25]. Curcumin is a natural phenolic compound with antitumor effects [26]. In this study, we found that curcumin inhibited HGF-induced proliferation *in vitro* and *in vivo* through EMT and that the mechanism is regulated by the c-MET-dependent PI3K/Akt/mTOR signaling pathways.

Meningioma is one of the most common malignant tumors of the central nervous system, accounting for approximately 13%–26% of all intracranial primary tumors [27]. Most meningiomas (78%) are benign (WHO grade I), 20.4% are atypical (WHO grade II), while 1.6% are malignant (WHO grade III) [28]. Most meningiomas are curable by surgery and radiation therapy; however, atypical and malignant meningiomas and even a few benign meningiomas are histologically or clinically aggressive and are prone to recurrence even after total tumor resection [29, 30]. The aggressiveness of meningioma is associated with aberrant activation of the EMT process [31]; therefore, EMT is a key therapeutic target for meningioma [32]. HGF is associated with tumor EMT. Moreover, HGF enhances the migration and invasive capacity of tumor cells [4, 24]. Curcumin has been shown to exert antitumor effects in various tumors [18, 33, 34]. It induces tumor cell apoptosis and inhibits tumor cell proliferation, growth, invasion, and anti-inflammation [18]. c-MET expression levels have been shown to be elevated in aggressive meningiomas, and curcumin inhibited the proliferation and migration of meningioma cells [20]. We investigated the association between curcumin, EMT, and HGF/c-MET in meningioma cells. We found that HGF and c-MET levels were upregulated in malignant meningioma clinical tissues, compared to benign meningioma clinical tissues, consistent with previous studies [20]. Curcumin has been shown to inhibit the proliferation and promote the apoptosis of benign human meningioma cells [21]. However, the effects of curcumin on human malignant meningioma cells have not been elucidated. We confirmed, for the first time, the inhibitory effects of curcumin on the viability of human malignant meningioma cells and established an optimal administration time and concentration. HGF enhanced IOMM-Lee cell proliferation, migration, invasion, and EMT while suppressing its apoptosis. Curcumin blocked these effects. In lung cancer [35, 36], non-small-cell lung cancer [37], liver cancer [38], and prostate cancer [39], HGF was found to induce EMT, thereby regulating tumor cell proliferation, migration, and EMT. In this study, curcumin treatment upregulated E-cadherin and downregulated vimentin and N-cadherin levels, which are associated with the EMT process. In accordance with these results, curcumin inhibited HGF-induced EMT in meningioma cells.

The mechanism through which HGF induces EMT is that, upon binding of HGF to its receptor, tyrosine kinase, c-MET is phosphorylated and subsequently activates downstream EMT-related signaling pathways [23]. Studies have shown that curcumin inhibits the progression of HGF-induced EMT in cells through different signaling pathways. For example, in lung cancer, curcumin inhibits EMT through the PI3K/Akt/mTOR signaling pathways [6], in renal tubular epithelial cells, it inhibits EMT through the PPAR*γ* pathway [40], in triple-negative breast cancer cells, it inhibits EMT through the TGF-*β* and PI3K/Akt signaling pathways [41], in human colorectal cancer, it inhibits EMT through the TGF-*β*/Smad2/3 signaling pathway [42], while in pancreatic cancer, it inhibits EMT through the PI3K/Akt/NF-*κ*B signaling pathway [43]. Activation of the PI3K/Akt/mTOR signaling pathway has been correlated with aggressiveness of human meningioma [13]. Therefore, we postulate that curcumin inhibits HGF-induced EMT through the PI3K/Akt/mTOR signaling pathway. However, the signaling pathway through which curcumin inhibits EMT in human malignant meningioma cells has not been established.

In this study, we found that HGF simulated the phosphorylation of cell surface receptor tyrosine kinase c-MET. Moreover, HGF induced the phosphorylation of c-MET, Akt, and mTOR. However, curcumin inhibited HGF-induced phosphorylations of c-MET, Akt, and mTOR in a concentration-dependent manner. The c-MET inhibitor (SU11274) and the PI3K inhibitor (LY294002) suppressed HGF-induced migration, invasion, and EMT of human malignant meningioma cells. These findings suggest that curcumin targets the c-MET and PI3K/Akt/mTOR signaling pathways to inhibit HGF-induced EMT in human malignant meningioma cells.

*In vivo,* we found that HGF was associated with increased tumor weights and sizes, while curcumin suppressed the HGF-induced increment in tumor weight and size. Immunohistochemically, HGF induction promoted EMT, while curcumin inhibited HGF-induced EMT in a concentration-dependent manner. These findings suggest that curcumin blocks HGF-induced tumor proliferation and EMT.

The efficacy of curcumin, used alone or in combination with other agents, has been evaluated in various clinical trials involving various diseases, including cancer, rheumatoid arthritis, Alzheimer's disease, diabetes, and leukoderma, among others [44]. Curcumin has been shown to block aggressive cancer progression by modulating various signaling pathways [34]. In this study, we found that curcumin suppressed HGF-induced migration, invasion, and EMT by inhibiting the c-MET/PI3K/Akt signaling pathway. However, we did not establish whether other signaling pathways are involved.

In conclusion, curcumin inhibits HGF-induced EMT by activating the c-MET-dependent PI3K/Akt/mTOR signaling. These findings suggest that curcumin is a potential therapeutic agent for malignant meningioma and provides an important basis for understanding the mechanism of action of curcumin.

## Data Availability

The datasets used and/or analyzed during the current study are available from the corresponding author upon reasonable request.
